# The role of the home health care physician in mobile integrated care: a qualitative phenomenograpic study

**DOI:** 10.1186/s12877-022-03211-3

**Published:** 2022-07-04

**Authors:** Lina Hovlin, Jenny Hallgren, Anna K. Dahl Aslan, Catharina Gillsjö

**Affiliations:** 1 School of Health Sciences, University of Skövde, P.O. Box 408, SE-541 28 Skövde, Sweden; 2 School of Health and Welfare, Jönköping University, Jönköping, Sweden; 3 Department of Medical Epidemiology and Biostatistics, Karolinska Institutet, Stockholm, Sweden; 4 College of Nursing, University of Rhode Island, Kingston, RI USA

**Keywords:** Home health care, Home health care physician, Integrated care, Person-centered care, Qualitative, Municipality care, Phenomenography

## Abstract

**Background:**

An increasing older population, along with the organizational principle of remaining at home, has moved health care from institutions into the older person’s home, where several health care providers facilitate care. The Mobile Integrated Care Model strives to provide cost-efficient, coherent, person-centered health care in the home. In the integrated care team, where the home health care physician is the medical authority, several health care professions work across organizational borders. Therefore, the aim of this study was to describe Home Health Care Physicians perceptions of working and providing health care in the Mobile Integrated Care Model, as well as perceptions of participating in and forming health care.

**Methods:**

A phenomenographic qualitative study design, with semi-structured interviews using an interview guide.

**Results:**

Working within Mobile Integrated Care Model was a different way of working as a physician. The physicians’ role was to support the patient by making safe medical decisions. Physicians described themselves as a piece in the team puzzle, where the professional knowledge of others was crucial to give quality health care. Being in the patients’ homes was expressed as adding a unique dimension in the provision of health care, and the physicians learned more about the patients by meeting them in their homes than at an institution. This aided the physicians in respecting patient autonomy in medical decision making, even though the physicians sometimes disregarded patient autonomy in favor of their own medical experience. There was a divided view on next of kin participation among the home health care physicians, ranging from always including to total absence of involving next of kin in decision making.

**Conclusions:**

The home health care physicians described the Mobile Integrated Care Model as the best way to work, but there was still a need for additional resources and structure when working in different organizations. The need for full-time employment, additional time or hours, more equipment, access to each other’s medical records, and additional collaboration with other health care providers were expressed, which could contribute to increased work satisfaction and facilitate further development of person-centered care in the Mobile Integrated Care Model.

**Supplementary Information:**

The online version contains supplementary material available at 10.1186/s12877-022-03211-3.

## Background

The organizational principle of remaining at home despite complex health care needs, along with the increasingly older population, has shifted the care of older persons from institutions to the home. Older persons receiving health care in the home often experience functional impairments, have several medical diagnoses, are frail, and require advanced medical and social care from different care providers [1–3]. Ageing is often challenging physically, psychologically, and socially. Older persons are reported to experience loneliness, which seem increase with several long-term conditions [4]. Hence, to support older patients’ and their next of kin’s needs to self-care, live well in their situation and participation in health care, despite health problems, different integrated care models have been implemented and researched in several places around the world [5–12]. Integrated care models bring together several professions in different constellations. The integrated care models could be interdepartmental with all the members of the team in the same organization [7], or collaborate across organizational borders [10, 13]. They might include municipality nurses working in collaboration with a patient’s family physician [12, 14] or with a patient’s primary care center physician [15]. Integrated care models have also been described as territory-based, where each geographic area develops its own version [6]. An integrated care approach can lead to a reduction in unnecessary hospitalization [16], faster response time to patient needs, better informed assessments, and pro-active identification of patient issues [14]. Integrated care can also lead to improved health and social care [11] and contribute to improved functional abilities and mental wellbeing in older persons [15]. However, integrated care can also lead to a heavier workload [14] and be challenging to implement if the local context is not attuned with integrated care. Other barriers found are lack of coordination, insufficient funding [17], and uncertainty about care responsibility [17, 18]. In Sweden, members of integrated care teams working in home health care are usually employed by different organizations. Several of the health care professionals are employed by the municipality, such as registered nurses (RNs) and occupational therapists, funded by the municipality. Physicians are employed by the primary health care, funded by the region, as this division of health care is stipulated in the law [19]. Hence, a collaborative way of working between the organizations is needed [20]. The Mobile Integrated Care Model (MICM) [21], is an example of a care model developed to increase collaboration and provision of coherent health care in the health care organizations in one Swedish region.

### Mobile integrated care model

**Fig. 1:**
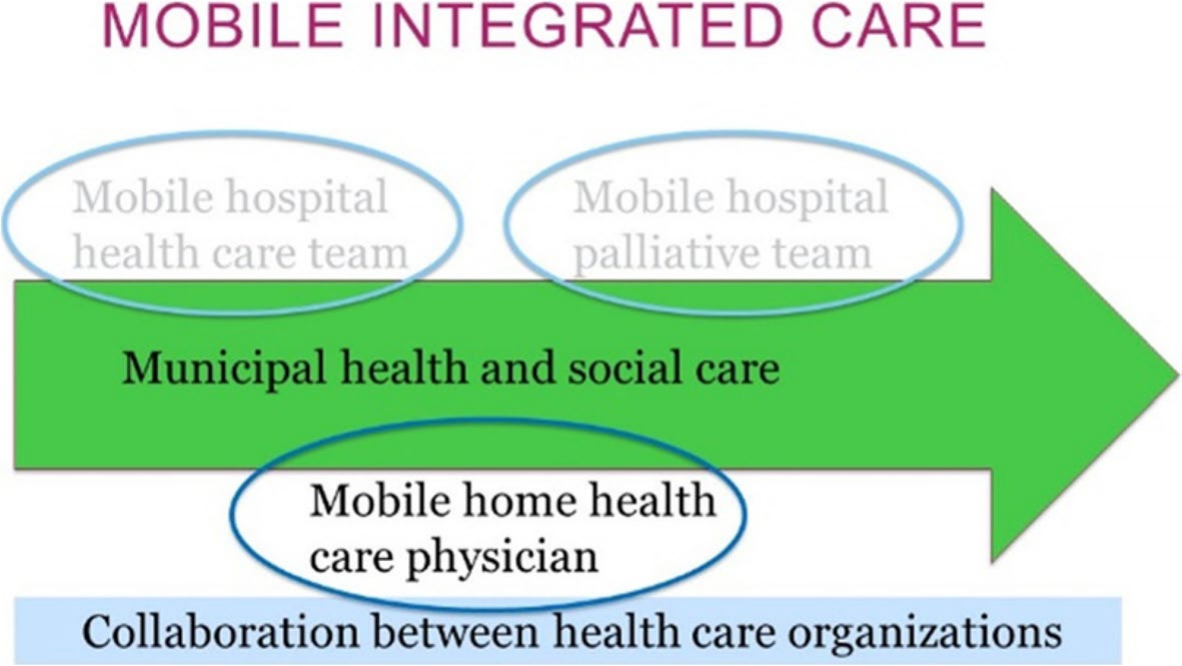
The Mobile Integrated Care Model

A comparatively new integrated care model, the MICM consists of three forms of health care: *the mobile hospital health care team*, *mobile home health care physician*, and *mobile hospital palliative team* (Fig. 1) [22] working in collaboration with municipal health and social care [21].

The MICM with a home health care physician is a collaboration between regional and municipal health care. The members of the integrated team employed by the municipalities are RNs, physiotherapists, occupational therapists, and assistant nurses (ANs). The physician, instead, is employed by the region through primary health care. The MICM includes:

- Having an appointed physician.
- The municipality RN and the physician making visits to an older person’s home.
- The integrated team is also to co-create a medical health care plan (MHCP) with the patient and their next of kin at least once a year.
- Beyond the included core elements within the integrated care model, the MICM has been implemented in varying ways in different municipalities [22].

Before the implementation of the MICM:

- There were no specific appointed physicians working toward home health care.
- All physician visits were done at the primary health care center. The patients rarely met a physician in the home.
- Different primary health care physicians, at times locum physicians, could be responsible for municipality health care each week.
- The appointed physicians were only responsible for home health care during set round times, often a couple of hours a week. If medical consultation was needed at other times, the municipality health care professionals often had to call the primary health care center and wait in line as the public did.

The model aims to provide cost-effective, quality health care to frail older persons living at home with complex health care needs [23]. The health care provided is to create quality of life for the patient and next of kin, and be coherent, despite different authorities having different areas of responsibility. The MICM focuses on health care rather than social care, even if health care professionals working in home health care collaborate with social care as well. The MICM is grounded in person-centered care [21, 24], which requires equal collaboration between the health care personnel and the patient [20] and is promoted by the World Health Organization [25]. Person-centered care [26, 27] stems from patient-centered care [28]. Physicians have historically worked patient-centered. Patient- and person-centered care have many similarities, but differ in aim. Patient-centered care aims to create a functional life for the patient within their diagnosis, while person-centered care focuses on creating a meaningful life for the patient outside of the care [29]. Person-centered care is described as having three cornerstones which builds a partnership between health care and the patient [27, 30]. The first is initiating the partnership by the patient’s narrative, which captures the person the patient is through the patients’ own narrative. The second cornerstone, working the partnership, involves creating a common to plan the care which should be designed from the patient’s own narrative through shared decision making. The third cornerstone, safe guarding the partnership, means documenting the patients’ preferences and involvement, legitimating patient perspective, and facilitating continuity [27] Person-centered care is not only based on who the person is, but furthermore the persons history, possibilities and obstacles they have in their current context. Patients are to be made active in their own health care and wellbeing, as well as gain a meaningful life outside of health care, rather than just within their health problems [27, 29–32].

The form MICM with mobile home health care physician will be the focus of this study. The MICM is based on collaboration between different professions, creating an integrated team where the physician mainly collaborates with the RN [33]. In a previous study, the nurses working in MICM described how collaboration was organized between them and that the physician was essential in how the quality of care was perceived, the nurses’ sense of work satisfaction as well as the sense of security of the nurses, patients, and next of kin [22]. To further describe other professions’ perceptions with the MICM will allow for a wider view of the model, where the physician has a major role. To our knowledge, few studies have focused on the physician’s perspective of working in an integrated team to provide coherent care across organizational borders, which can highlight the physician’s perspective for similar integrated care models. These aspects make for a highly interesting study subject.

### Aim

To describe home health care physicians’ perceptions of working and providing health care in the Mobile Integrated Care Model, as well as perceptions of participating in and forming health care.

## Method

A phenomenographic [34], qualitative study design was conducted, with semi-structured interviews using an interview guide, to describe the phenomenon. The study was designed with the purpose of gathering data about physicians’ perceptions working within the MICM. The design provided the opportunity to gain access to the physicians’ varied perceptions, while also gaining depth through follow-up and probing questions. Phenomenography is an empirical research approach developed by Marton [35, 36], which qualitatively describes various ways in which different phenomenon in the world are experienced, conceptualized, understood, and created. The different ways of understanding the phenomenon are conveyed through an outcome space consisting of qualitatively different categories. The outcome space is the range across the variation of perceptions in how the phenomenon is understood, as well as the structure and hierarchic order of the qualitatively different categories.

### Participants

Primary health care managers of two primary health care territories as well as unit managers at two private primary health care centers were asked for their consent to allow home health care physicians in their organizations to participate in an interview. All agreed to participate. These cover the 15 municipalities where the MICM was first implemented. The project manager asked unit managers at primary health care centers in the 15 municipalities if they would participate. Three declined participation, since they did not have the preconditions to work according to the MICM and one did not respond. Physicians in the remaining 11 municipalities who had worked as home health care physicians for at least six months were asked by their unit managers if they wanted to participate. If the physician accepted, the unit manager sent contact information to the project manager or gave permission to the project manager to directly contact the MICM-physicians and ask if they wanted to participate. Eighteen physicians were asked to participate, and seventeen agreed. One physician who had initially agreed did not participate due to outside circumstances, leaving 16 participating physicians. The participating physicians ranged in age from 37 to 68 years. Ten were male and six female, with work experience varying from 12 to 45 years. The majority were district physicians, while some had a specialty in internal medicine or geriatrics.

### Data collection

Semi-structured interviews were conducted by three of the researchers as well as two students in a postgraduate specialist nursing program in primary health care nursing. The interviews lasted between 20 and 72 min and were recorded and transcribed into text. Field notes were made by the researchers after each interview but were not included in the analysis. The interview guide was constructed with open-ended questions. The questions allow a participant the freedom to choose the dimensions of the phenomenon as they perceive it. Furthermore, it creates space for unexpected answers to help the researcher understand the whole [37]. Two pilot interviews were conducted to test the interview guide and were deemed satisfactory in addressing the aim of the study. The pilot interviews were therefore included in the results. Ten interviews were conducted during the winter of 2018–2019, and eight were conducted during the autumn of 2020. This was done to include more municipalities in the data. The interviews took place at a location chosen by the physicians. Fourteen chose their workplaces, while two preferred their homes. Twelve were done in person, while four were done digitally.

### Data analysis

The study’s data analysis was conducted according to the phenomenographic method by Sjöström and Dahlgren [34] with origins in Dahlgren and Fallsberg [38]. The method of analysis consists of seven steps. The process of analysis is not linear, but iteratively, where the different steps of analysis are repeated as necessary. First, the interviews were listened to and read through several times for the researchers to *familiarize* themselves with the data. Three of the four authors conducted the interviews, and all read the transcripts to become familiar with the text. Corrections of errors in transcriptions were also made. In the second step, *compilation*, significant elements perceived by each participant in relation to the aim were identified. A total of 759 significant elements that were identified were *condensed* in the third step to find the central parts, the core, in the informants’ answers. In the fourth step, *grouping*, condensed data, which showed similarities, were preliminarily classified into 19 groups. The condensed data were used to identify similarities and differences to establish borders between the preliminary categories. *Comparison* of the preliminary categories was done in the fifth step of the analysis, and resulted in six descriptive categories. In step six, the categories were given *names* to emphasize their core. In the seventh and final step, the *constative comparison* was described and organized in the outcome space. The outcome space constitutes the descriptive categories describing the qualitatively different ways in which the phenomenon was understood and how the categories relate to each other. The analysis was done through discussions in the research group, frequently, and through several rounds, until agreement, a negotiated consensus, [39] was reached. The researchers’ preunderstanding of the studied phenomenon was restrained in the process of collecting the data, as well as through the analysis, to reveal the different perceptions the participants had toward the phenomenon [40].

### Ethical considerations

The project was approved by the Swedish Ethical Review Authority (Dnr 1020-17; 2019–02563; 2020–04324), and conducted according to the ethical guidelines of the Declaration of Helsinki [41]. All informants received written and oral information about the aim of the study and that participation was voluntary, meaning they could exclude themselves from the study at any given time without subsequent consequences. Informed consent was obtained from all participants.

## Results

The following six qualitatively different descriptive categories constitute the results of the study. The descriptive categories reflect the different variations of the phenomenon, physicians’ perceptions of MICM with home health care physician, as well as perceptions of participating in and forming health care. The descriptive categories form the outcome space, presented at the end of the results.

### A different way of working as a physician

The physicians described being a home health care physician as a different way of working, which was flexible, enjoyable, and exciting. One physician said: *“It’s a dream come true, it’s amazing, very rewarding, it’s fun and you feel like you do a lot of good and create a sense of safety.”* It was expressed as the best way to work, even if providing health care for severely ill older persons could be perceived as draining. The continuity and accessibility that the physicians could offer was perceived as bringing quality health care to the patients. The physicians described the MICM as the future of care, and struggled to find downsides to it. However, some expressed that the role of a home health care physician could become lonely. To avoid the feeling of isolation, several physicians kept an office at the primary health care center, as well as participated in meetings with the other home health care physicians working in the region.

The different way of working as the home health care physician involved being responsible for the medical care of the patient. Their role in supporting the patient’s health was, according to the physicians, making sure they did not endanger the patient through unnecessary medication, causing troublesome side effects. The role as a home health care physician was also to be pedagogical in explaining why a medication was to be prescribed or removed, and why the patient might be feeling the way they were. Some physicians described that the different way of working did not involve giving health advice or creating meaningfulness. One physician expressed: *“I don’t think in terms like that, well-being is too wide for me. I work with the medical sense of safety and handling.”* Another physician acknowledged that they were employed for their medical knowledge, but that their role extended beyond that, and said: *“I try to keep an overall perspective. I’m employed to have opinions on the medical, but it’s about the person and the history too.”* The general opinion among the physicians was that their role was to focus on the medical side and creating a sense of security, making safe medical decisions, and supply continuity.

### A piece in the team puzzle

Being a home health care physician entailed being a part of something greater than themselves. One physician said: *“I feel like a piece in a puzzle.”* The physicians claimed that the team-based work within the MICM elevated the quality of care, since different professions brought different perspectives. One physician expressed: *“As a doctor I’m not all-knowing, that’s why we have different professions.”* In some municipalities, the physician met all the health care professions regularly and appreciated their competence. Other physicians viewed their part in the puzzle to mainly collaborate with the RNs. It was the RNs’ role to convey information from the physician to other professions. The physicians often referred to themselves in one of two roles within the team. One role was described as the spider in the web, which meant being the center of the team, the medical treatment, and the decision making surrounding the patient. This required help from the other professions. *“I’m the spider in the web, but without them I couldn’t work,”* one described it. The other physicians viewed themselves as consultants, where the municipality health care commissioned their time and competence. One physician expressed, *“This is a consulting business; they buy this service from us, my time.”*

The collaboration with the RNs working in the MICM was described as good by the physicians. The RNs had closer contact with the patients according to the physicians, so their perspective of the patients was valued by the physicians. The collaboration worked best if the physician and RN knew each other, and building that relationship took time. The physician perceived that having an office in the same corridor as the RNs facilitated good communication, and made the work easier. Preserving knowledge and competence by being an educator on the team was expressed as valuable, and was seen by the physician as one of their pieces in the puzzle. Several physicians described wanting to visit conferences or hospital wards together with the RNs to elevate the competence of the team as well as to do a teambuilding activity.

The physicians described the ANs as a resource in the team, and the physicians found it good when they joined the home visits. *“The assistant nurses are the ones that see the patient every day and their role is to help them with the everyday things. It’s really important that we can talk to them who are the closest support for the patient,”* one physician said. In some municipalities, the ANs were not able to join in on home visits because their schedule was too busy, which the physicians described as unfortunate.

### Being in the patient’s home adds a unique dimension

The physicians described that being in the patient’s home opened a different dimension compared with seeing the patient at the primary health care center. Being in the patient’s home was described as having positive aspects by all physicians. One physician said: *“You get a unique opportunity to see a person in their home environment, in their home arena. It’s a different dynamic there.”* The physicians described how indirectly they were able to gather a lot of information from being in the home, for example, how the patient moved and how they stored their medications. It was described as a more person-centered way of care. Additionally, the physicians perceived that the patients followed their prescriptions and understood their explanations better when these were given in the home. Other physicians had not considered the sense of home for the patient. One said: *“I don’t know if I should think too much about if they feel at home, sometimes many visits are needed and it’s almost a hospital, a lot of staff and equipment.”*

The physicians described themselves as visitors in the unique home arena, a guest who needed to adapt to the patient’s wishes and routines. The physicians had to watch the way they behaved and not overstep. The physicians had different views on being social, and some said it was not their role, while others claimed the opposite. *“I come with a purpose, not to drink coffee. I come to gain a view on how they feel and how to help them,”* one described, while another said: *“They’ve baked and I always accept because otherwise it’s disrespectful. You get closer to them that way.*” The home visit was a more relaxed form of meeting with the patient. The home environment opened up for several persons—patient, next of kin, and health care professionals—to express their point of view about the patient’s situation. The patients told the physicians that it was a luxury to have a physician in their home. The benefits of being in the patient’s home were also compared with providing health care at the primary health care center. The latter was described as draining for the patient. One physician explained: *“The severely ill persons who I work with, it’s hard to have good conversations with them at the primary health care center. They’re often tired and they don’t have the energy to bring their life to the primary health care center and back. It’s better I go to them, where the problem is.”* Therefore, physicians saw it as beneficial for them as well as the patients that they visited in their homes.

### Respecting patient autonomy in medical decision making

The physicians described it as central that the patients decided for themselves which of the given treatment options they preferred. The physicians tried to listen to the patients’ expectations, explained, and informed, and then let them make the decision themselves, which was later documented in the MHCP. The physicians expressed that even though it was a collaboration with the patients, the physicians were the medical authority and had the final say in medical decision making. One physician said: *“On the one hand you want to respect the patient’s wishes, but on the other hand, as a physician you know medically what would be good.”* The physicians created a sense of security for the patients by being well-read, explaining, and being open with medical records, such as the MHCP. The physician sometimes had to bring up tough questions and approach subjects, such as moving to an assisted living facility or death, which were not always easy to have a dialogue about. They perceived that their honesty about difficult topics created security. They also shared that respecting the autonomy of the patients and allowing them to participate did not entail that the patients had to decide everything. The physician was still the medical authority on medical decisions. At other times, the patients did not want to make choices, and the physicians felt it was their responsibility to respect those choices, and made the medical decisions for the patients. The physicians found it important to consider the patients’ autonomy, but found it was not always possible when making safe medical decisions.

### A divided view on next of kin participation

The physicians expressed differing views on next of kin participation in the MICM. Some believed that next of kin should not participate in decision making at all. Others found next of kin to be a good resource in decision making and always wanted them to be informed and participate. One physician said: *“Next of kin do not participate, they shouldn’t.”* Another expressed: *“My role is to inform next of kin about the collaboration, so they feel like they’re participating. Maybe not making decisions but being informed.”* A third view was: *“We try to involve next of kin as much as possible.”* The physicians stated that the patients needed to approve contact with the next of kin so that the physicians could feel comfortable sharing information with them. In many physicians’ opinions, open communication and being a part of the decision-making process made the next of kin feel safer. If the next of kin could not participate in the home visit, physicians said that they or the RN called the next of kin and informed them of the decisions afterwards. According to the physicians, it was mainly the RN’s role to be in contact with the next of kin. Sometimes the next of kin did not agree with the physicians’ decisions or had what the physicians described as unreasonable requests, which created conflicts. In these situations, the physicians were clear that the patient was their main focus, and the patient’s opinion along with their own was what guided the physician when making a decision. The physicians had contrasting views on next of kin participation in the MICM, and the contact with the next of kin differed because of this divided view.

### Need for additional resources and structure when working in different organizations

Because the allocation of resources differed, the home health care physicians had varying percentages of employment within the MICM. The implication was that for some teams, there were times when there was no home health care physician available when there was not one employed full time in the MICM. The division of time and the different organizations impacted the communication and accessibility to the physicians for the RNs. This was expressed by the physicians as a quality risk. They pointed out that this was a political and organizational decision that they had little opportunity to influence. When the physicians were not available, a robust MHCP was considered to be helpful and made the RNs and patients feel safer. Time was a recurring resource that was brought up by the physicians. While some expressed they had enough time, most physicians perceived that having an increase in hours in the function as home health care physician was their main objective in improving the MICM. They believed an increase in hours would give them the opportunity to conduct more home visits to acute cases and perhaps prevent hospitalizations. The lack of time led to prioritizing, or the physicians had to work overtime because of the heavy workload and unwillingness to disregard patient safety.

An on-call schedule between the home health care physicians in the region was suggested as a possible improvement. It would provide an opportunity for the RNs to call a home health care physician outside of regular business hours instead of a physician who did not understand the MICM. Other suggestions were a small geriatric ward in each city, or that the home health care physician could admit patients to a geriatric hospital ward or to the municipal short-term care accommodation. The physicians also wished for closer collaboration with the other MICM teams, the mobile hospital health care team, and the hospital palliative care team.

Medical records were seen as an area in which the physicians wanted to see improvements. The physicians described how the RNs did not have access to the physicians’ medical records, and the physicians did not have access to the municipality professionals’ medical records. The physicians stated that the different medical record systems resulted in double work, since information needed to be added to both. The lack of equipment within the MICM was something the physicians saw as another possible way to improve the model. One said: *“More equipment. I borrow from the primary health care center, but they prioritize themselves, so it’s hard to get a hold of.”* They described that added equipment would lower the need to call an ambulance and aid in determining which treatment the patient should receive. The equipment suggested by the physicians was medical, but also digital, for example, access to a medical record while they were in a patient’s home.

### Outcome space

The descriptive qualitative categories constitute the outcome space, in which the internal relationship and hierarchy between categories are described. The first five descriptive categories: *A different way of working as a physician, A piece in the team puzzle, Being in the patient’s home adds a unique dimension, Respecting patient autonomy in medical decision making*, and *A divided view on next of kin participation*, represent the range of how the physicians perceived working with the MICM. All categories are main categories. The first, *A different way of working as a physician*, involves the home health care physicians’ own views on their role and how they perceive it. This category is viewed to be higher in hierarchy than the others, since it describes the physicians’ views about their role as a different way of working as a physician, which affects and permeates the way they perceived the phenomenon and express themselves. *A piece in the team puzzle* involves the physicians’ role within the team and their views on collaborating with other personnel. *Being in the patient’s home adds a unique dimension* involving the physicians’ perceptions of a changed perspective of their role as physician by being in the patient’s home. *Respecting patient autonomy in medical decision making* involves the physicians’ reflections about the patients’ autonomy in medical decision making. *A divided view on next of kin participation* entails the physicians’ differing conceptions of next of kin involvement in health care. The second part of the aim of this study, the physicians’ perception of participating in and forming the health care, is represented in the final descriptive category, addressing the need for improving the MICM, named *Need for additional resources and structure when working in different organizations*.

## Discussion

The physicians described how they enjoyed the role of home health care physician. Working in the MICM was perceived as improving quality in the health care provided to older patients. This view of the model is also supported by RNs, who stated that they never wanted to go back to the way they worked before the implementation of the MICM [22]. Similar perceptions have been expressed by other health care professionals working with older patients in integrated teams. It was also expressed that the work with the older persons was rewarding, as well as challenging and draining, which is also supported by other studies [42]. The role of the home health care physician was described in the current study in two different ways: as a spider in a web and as a consultant. Nurses working in the MICM have also described how the home health care physician was a consultant in the MICM, explaining that the physician became an outsider in the teamwork [22].

According to the physicians, the integrated team in the MICM was an important component in the provision of health care because of the different viewpoints provided by the different professions. The need for competence from different professions in home health care has been described previously [18, 42], as the complex care needs of the patients expose the limitations of the physicians’ health care [18]. It was evident from the interviews that the physicians focused on the medical care, which is of course also in line with their profession and responsibility in the team, and few viewed their role to focus social contexts or the patients’ life outside of health care.

There are also challenges with teamwork in integrated teams employed by different organizations. The lack of willingness to share information between organizations as well as a low level of respect from physicians toward municipality staff has been described by those working in the municipality [9]. Such findings were not supported in the current study. Rather, the results relate to other studies on integrated care, where the importance of joined care meetings and good communication was expressed [7, 22]. The physicians in the current study wanted better opportunities to share information between organizations and to continue to strengthen the team through joined team activities. They described the competence of the other professions as valuable to the team to create quality care for the patients in the home.

In earlier research, the home was described as a place that reflects the older person’s values, beliefs, personality, and way of life [43]. In the current study, the physicians described that being in the home of the patient gave them an understanding of who the patient was and the life they lived. However, some physicians felt that physicians should not think about whether the patient felt at home, since changes to the home space were sometimes considered necessary to maintain quality care. Autonomy over the home space has been defined as fundamentally affecting the sense of home [44], which might be disturbed when home health care providers enter or expand their role in the older patient’s home [43]. The need to address the older person’s autonomy and way of living in the provision of health care in the MICM is therefore of significance to not disturb the sense of home and to strive for person-centered care, which the MICM aims to provide.

The MICM aims to provide person-centered care [21, 24, 26]. Initiating the partnership is the first of the three cornerstones of person-centered care [27], which was something the physicians struggled with. The physicians expressed that being in the home was a way to work person-centered, since they saw who the patient was and how they lived. The current result is supported by previous research stating that if physicians do not make home visits in integrated care, they work less person-centered, and are not able to understand the patient’s everyday life [12]. Health care professionals working in integrated teams have described as an important aspect for successful collaboration that the physician has a holistic view of the patients and not only the medical diagnoses [7, 22]. Some of the physicians in the current study expressed that their role was *not* to see beyond the medical aspects, while others expressed a wider view of their role where the patient’s history and who they were as a person. It is acknowledged that social ties with ones’ community can increase well-being for older persons [45]. From a person-centered care perspective, it is therefore interesting that the physician viewed their role in the integrated care to mainly focus on the medical aspects of the patients’ situation. Obtaining patient perspectives on MICM may show where social support is given. It is therefore not fully possible to claim the care model is or is not person-centered based on solely the perspective of the physicians, and a wider view from several perspective is needed.

The physicians described that they listened to the patient’s expectations and then let them make decisions themselves, trying to respect their autonomy. This can be viewed as working the partnership, which is the second cornerstone of person-centered care [27]. However, respecting patient autonomy was sometimes expressed as challenging. The physicians described how the patient’s narrative was sometimes overlooked when decisions for medical treatment were made. It has been suggested previously that general practitioners (GPs) have different practice patterns when working with older persons health care goals [46]. In one pattern, GPs try to convince their patients of what the medical goal should be, rather than having the patient express their own personal goals. This practice pattern was described by some of the physicians in the current study and is not consistent with the person-centered approach. Another practice pattern described how a patient’s goals were important, but that the GP had a strong focus on avoiding risk and ensuring safety for the older person [46]. It was evident in the current study that several of the physicians shared this view. This practice pattern can be viewed as working in a partnership, where both the patient and the integrated team express their views and discuss what type of health care goals to continue working toward.

The third cornerstone of person-centered care is safeguarding the partnership through documenting the patient’s narrative [27]. The patient’s requests and views were documented through the MHCP. Some physicians expressed that lack of time prevented them from updating the MHCP as frequently as they needed, an issue RN working in MICM also mentioned [22]. The complexity of caring for persons with multiple long-term conditions indicates a need for more time for health care professionals to meet the health care needs of the patients [42]. In the current study, the physicians described working overtime so as not to compromise patient safety. To achieve person-centeredness, updated, structured person-centered health care plans are needed [30]. The MHCP can therefore be seen as valuable for physicians when working toward person-centered care. Full-time employment within the MICM, as well as a connected system for medical records, were also suggested as possible organizational improvements to advance quality of care within MICM. The lack of joined medical records [17, 22, 42] and resources has been previously described as one of the development opportunities in integrated care models [17]. It is known that person-centered care has organizational barriers, such as time constraints, as well as challenges within medical records [30]. To facilitate person-centered care, as the MICM is striving for [21, 24], the suggested improvements from the physicians should be acknowledged by the responsible health care organizations to ensure safe and high quality health care for the older patients.

Beyond the suggestions for improvements mentioned by the physicians, it is also evident from the interviews that some physicians struggled more than others with providing person-centered care. Hence, it can be interpreted that rather than the MICM being person-centered, it depended on the physician whether he or she had a person-centered approach. It should be mentioned, that despite that some physicians might not have expressed that they had a person-centered approach, other team members might provide person-centered care.

### Strengths and limitations

In the current study the focus is on the perceptions of MICM from the MICM-physician perspective, while we in a previous study reported the RN experiences of MICM [22]. Both studies are a part of a larger project, studying MICM from the perspectives of patients, next of kin and health care professionals. In both the previous and the current study semi-structured interviews are used. This can be seen as a limitation since the extent of which generalizations feasibly can be drawn may differ depending on hidden biases within the participation group. Interviews gain a deeper understanding of the participants’ experiences, but might lead to the respondents answering what they think the interviewer wants to hear or what is politically correct. Accordingly, future research could consider the use of other methods, like for example observations, to add a source of data collection for increased understanding of the strengths and limitations of MICM. A triangulation through mixed method could also have viewed the role of the MICM-physician from several perspectives, which could have exposed hidden biases which could have been expressed in the interviews. However, studying MICM from several perspectives may led to a broad view of all roles within the care model, since the participants describe their own view of their work and role as well as their colleagues work and role in the team in MICM.

This study was conducted by a team of researchers, and while one researcher was responsible for exploring the different conceptions of the phenomenon, the content and the category names were discussed within the full research team. Discussions were conducted several times, at different moments, until agreement was reached to create a negotiated consensus, and described to enhance the trustworthiness of phenomenographic research [37, 39]. The analysis [34] was iterative, where the seven different steps were repeated if needed, and not followed linearly. Each new read-through formed a new understanding of the material, and these new perspectives were then explored with reference to the entire pool of data. To increase credibility, the method section of the study was detailed in the description to show all the steps of the research process [37]. The quality of the outcome space can be viewed in light of how the descriptive categories were identified, are qualitatively different, and show the differences in how the phenomenon was perceived. The descriptive categories are logically related through the perception of the home health care physicians, and their interrelationship can be viewed in the outcome space. Another criterion for quality is presenting findings in as few descriptive categories as possible [47]. The outcome space consists of six descriptive categories, which can be viewed as many. However, the aim of this study was multifaceted, with two distinct parts, which motivated the number of descriptive categories in order to present qualitatively different ways of perceiving the phenomenon in a defensible, useful, and meaningful way to the intended audience [48, 49].

## Conclusions

The home health care physicians described that working within the MICM was a different way of working as a physician than they were used to. Their role as a home health care physician was to support the patient by making sure to make safe medical decisions and to avoid troublesome side effects. The home health care physicians described themselves as a piece in the team puzzle, where the professional knowledge of others was crucial to give quality health care to the patient within the home. Being in the patient’s home was described as adding a unique dimension to health care, and the home health care physicians learned more about the patient when meeting them in the home than at the primary health care center. This aided the home health care physicians in respecting patient autonomy in medical decision making, even though the physicians sometimes disregarded patient autonomy in favor of their own medical experience or opinion. There was a divided view on next of kin participation among the home health care physicians. Some included the next of kin in all decision making, and others not at all. The home health care physicians described the MICM as the best way to work, but there was still a need for additional resources and structure. Full-time employment, additional time or hours, more equipment, access to each other’s medical records, and additional collaboration with other health care providers were needed, which could contribute to increased work satisfaction and facilitate further development of person-centered care in the MICM.

## Supplementary Information

**Additional file 1.**

## Data Availability

The datasets analysed during the current study are not publicly available due to ethical principles and the Swedish Ethical Review Authority but data not comprising confidential information are available from the corresponding author on reasonable request.

## Abbreviations

MICM: Mobile Integrated Care Model
RN: Registered nurse
AN: Assistant nurse
MHCP: Medical Health Care Plan
