# Supplementary material for: The role of the home health care physician in mobile integrated care: a qualitative phenomenograpic study
Source: BMC Geriatr. 2022 Jul 4;22:554. doi: 10.1186/s12877-022-03211-3 (PMC9252081; doi:10.1186/s12877-022-03211-3)
Supplement: Supplementary file 1 — Additional file 1. [file 12877_2022_3211_MOESM1_ESM.docx]

**What perceptions does physicians have of mobile integrated care model?**

**Demographic data:**

Age, gender, profession, active years within the professions, previous professions?

***Tell me about your perception of providing health care in the care model mobile integrated care?**

***How do you perceive the collaboration: assistant nurses, home health care professionals, nurses, home health care physicians.** *Is there anything you would like to change? What?

***What support can you give patients and next of kin for them to feel good in their daily life?**

*How is health and well-being supported? (physical, phycological, social)

*How is meaningfulness supported in the care provided?

*How is autonomy supported in the care provided?

*How are the patients’ own abilities supported in the care provided?

***In what way do you adjust your care towards the patient and next of kin’s needs?**

*What does it look like?

*How could it be improved?

***In what way do you initiate patient and next of kin participation in the health care you provide?**

*What does it look like?

*How could it be improved?

***How are patients and their next of kin involved in developing health care?**

*What does it look like?

*How could it be improved?

***What ethical aspects can you see in the health care provided towards the patients and next of kin surrounding participation?**

*What does it look like?

*How could it be improved?

***How do you perceive the quality of the health care provided?**

*How do you perceive the work with maintaining and developing the quality of care?

***What do you do to make patients and their next of kin feel safe?**

***Describe what you do to make patients feel: Well-being? Feeling at home within the home?**

***What effect do you think mobile integrated care model has on the patient and next of kin’s sense of: Well-being? Home?**

***Are there correlations to the sense of home and the sense of well-being?**

*In what way?

***What effect has the COVID-19 pandemic had on your work in mobile integrated care model?**
